# Incidence and Risk Factors of Acute Kidney Injury after Kasai Operation for Biliary Atresia: A Retrospective Study

**DOI:** 10.7150/ijms.44163

**Published:** 2020-04-06

**Authors:** Jin Ha Park, Kyong Ihn, Seok Joo Han, Sijin Kim, Sung Yeon Ham, Sangmin Ko, Min-Soo Kim

**Affiliations:** 1 Department of Anesthesiology and Pain Medicine, and Anesthesia and Pain Research Institute, Yonsei University College of Medicine, Seoul, Republic of Korea.; 2 Department of Pediatric Surgery, Severance Children's Hospital, Yonsei University College of Medicine, Seoul, Republic of Korea.

**Keywords:** acute kidney injury, biliary atresia, epidemiology, Kasai operation, risk factors

## Abstract

**Background**: Biliary atresia is a progressive, inflammatory, and destructive pathology of the bile ducts. Patients who undergo surgery for correction of biliary atresia (Kasai operation) are at risk of acute kidney injury (AKI) because of their young age at the time of surgery, long operation time, and liver fibrosis or failure as complication of biliary atresia. Conversely, AKI is associated with poor outcomes after surgery. This study therefore aimed to evaluate the incidence, risk factors, and outcomes of AKI after Kasai operation.

**Methods**: All consecutive patients who underwent Kasai operation between March 2006 and December 2015 in a single tertiary-care university hospital were enrolled. AKI was defined based on the Acute Kidney Injury Network criteria. Multivariate logistic regression models were used to assess risk factors for AKI.

**Results**: One hundred sixty-six patients received Kasai operation during study period. Of these, AKI occurred in 37 of 166 patients (22.3%). In multivariate logistic regression analysis, age older than 30 days, higher preoperative estimated glomerular filtration rate, and preoperative contrast use within 7 days were associated with the development of AKI. Perioperative packed red blood cells transfusion was related to reduced occurrence of AKI. AKI was associated with longer ICU stay (OR = 1.015, p = 0.016). More patients with AKI were also found to receive additional surgery except liver transplantation within 1 year compared to those without AKI (10.8 % vs. 2.3 %, *p* = 0.045).

**Conclusions**: Increased age is strongly associated with the development of AKI after Kasai operation. These findings indicate a rational basis for early corrective surgery for biliary atresia, early screening for AKI, and intervention to improve the results of Kasai operation.

## Introduction

Acute kidney injury (AKI), even when mild and transient, is increasingly acknowledged as a risk factor for poor prognosis, including longer duration of hospital stay, mortality and morbidity in children.^1^-^3^ Of note, even a minor post-operative rise in serum creatinine is associated with a poor outcome in both children and adults.^4^,^5^ Despite the advances in understanding of its pathophysiology, the disease pattern and spectrum of AKI remain unclear, and effective preventive measures for the disease still remain unproven. Moreover, most previous studies regarding pediatric AKI have focused on patients underwent cardiac surgery or critically ill patients.^1^,^3^,^6^ In that regard, information on epidemiology and outcomes of AKI in pediatric population is scarce.

Biliary atresia is a progressive, inflammatory, and destructive pathology of the bile ducts. Although its long-term outcome is controversial and more than half of patients with biliary atresia are registered on waiting lists for liver transplantation,^7^ Kasai operation is suggested as the primary treatment for this condition.^8^ Children undergoing Kasai operation are at risk of AKI because of their young age and the associated immaturity of their kidney function at the time of surgery, frequent exposures to contrast agents and nephrotoxic drugs, long operation time, hemodynamic instability during surgery, and co-existing liver fibrosis or failure as a complication of biliary atresia.^9^,^10^

The present study aimed to describe the incidence and risk factors of AKI after Kasai operation and to determine whether AKI affects the outcomes of patients after Kasai operation.

## Methods

### Study design and data collection

This study was conducted after approval from the institutional review board (IRB number 4-2017-0496) of the Severance Hospital. Informed consent was waived due to the retrospective nature of the study. We reviewed 166 consecutive patients with biliary atresia who underwent Kasai operation performed by a single surgeon (S.J.H) at the Severance Hospital, Seoul, Korea, between March 2006 and December 2015.

Preoperative variables that were considered included patient demographics; preoperative laboratory data, including serum creatinine, estimated glomerular filtration rate (eGFR) by updated Schwartz equation,^11^ serum hemoglobin, serum direct bilirubin, and aspartate aminotransferase (AST)-to-platelet ratio index (APRi); Pediatric End-Stage Liver Disease (PELD) score; and contrast use <7 days prior to the operation. APRi is calculated as AST level (IU/L)/upper normal limit (50 IU/L) × 100/platelet count (10^3^/μL). Intraoperative and postoperative variables considered included anesthetic time, type of anesthetic, use of antibiotics, use of vasoconstrictors, fluid balance, blood transfusion, lowest blood pressure, need for intensive care unit (ICU) admission or mechanical ventilation, and any additional operation except liver transplantation within 1 year.

### Definition of AKI

The primary end point was the incidence of postoperative AKI as defined by the Acute Kidney Injury Network (AKIN) criteria,^12^ which has been widely used in previous pediatric studies.^1^,^13^ Preoperative serum creatinine level was used as the baseline. AKI was defined as an absolute increase in serum creatinine level of at least 0.3 mg/dl from preoperative baseline or at least 50 % increase in serum creatinine concentration within the first 48 hours after surgery. Considering the retrospective design of the study, if there was no serum creatinine result available at 48 hours after surgery, the next serum creatinine level within the first 5 days after surgery was used.

### Statistical analyses

Continuous variables were expressed as means ± standard deviation or median (Q1-Q3), while dichotomous variables were expressed as percentages. Continuous variables were compared using independent Student's *t* test or Mann-Whitney *U* test, and dichotomous variables were compared using chi-square or Fisher's exact test as appropriate.

Backward stepwise logistic regression analysis was performed to evaluate the independent risk factors for AKI. Variables used in the final multivariate model were selected performing until no further variables can be deleted on a criterion of p<0.20 in the first multivariate model which involves starting with all candidate risk factors to be predictive of AKI. Therefore, variables used in the multivariate logistic regression model included the following variables: age >30 days, preoperative eGFR calculated by the Schwartz formula,^11^ preoperative contrast use within 7 days, and perioperative packed red blood cells (pRBC) transfusion. The model was adjusted for the following prognostic factors: length of ICU stay and survival with native liver (SNL). Cut-off value of age >30 days was determined based on the previous studies that showed jaundice disappearance rate was higher in patients undergoing Kasai operation before 30 days after birth.^14^-^16^

The Kaplan-Meier method was used to evaluate survival rate. SNL and patient survival were compared according to the occurrence of AKI. SNL ends at liver transplantation or at death in patients who did not receive liver transplantation.

Data analysis was done with SPSS 25 (SPSSFW, SPSS, IBM, Armonk, NY, USA). *P* values less than 0.05 were considered statistically significant.

## Results

The baseline characteristics and perioperative data of the patients are presented in Table 1 according to the occurrence of AKI. Of the 166 patients enrolled, 37 (22.3%) developed AKI. Compared with patients in the non-AKI group, patients in the AKI group were significantly older (median age 73 vs 58 days, *p* = 0.009) and had significantly higher eGFR (126 vs 86 ml/min/1.73 m^2^, *p* <0.001) at the time of surgery. More patients in the AKI group were older than 30 days, compared to the non-AKI group (97.3 % vs. 82.2%, *p* = 0.021). Intraoperative and postoperative variables assessed at 48 h were comparable in both groups. However, more patients in the AKI group (10.8% vs. 2.3 %, *p* = 0.045) received additional surgery apart from liver transplantation (such as adhesiolysis or small bowel resection) within 1 year. The number of patients who underwent liver transplantation and the number of patients who died within 1 year were similar between the groups.

Analysis of risk factors for postoperative AKI after Kasai operation is shown in Table 2. In this multivariate regression analysis, age >30 days (odds ratio (OR) = 22.711, *p* = 0.041), preoperative eGFR (OR = 1.020, *p* = 0.001), and preoperative contrast use within 7 days (OR= 5.513, *p* = 0.044) were associated with the development of AKI. Perioperative pRBC transfusion was associated with reduced occurrence of AKI (OR = 0.284, *p* = 0.014). AKI was associated with longer duration of ICU stay (OR = 1.015, *p* = 0.016).

**Figure 1:**
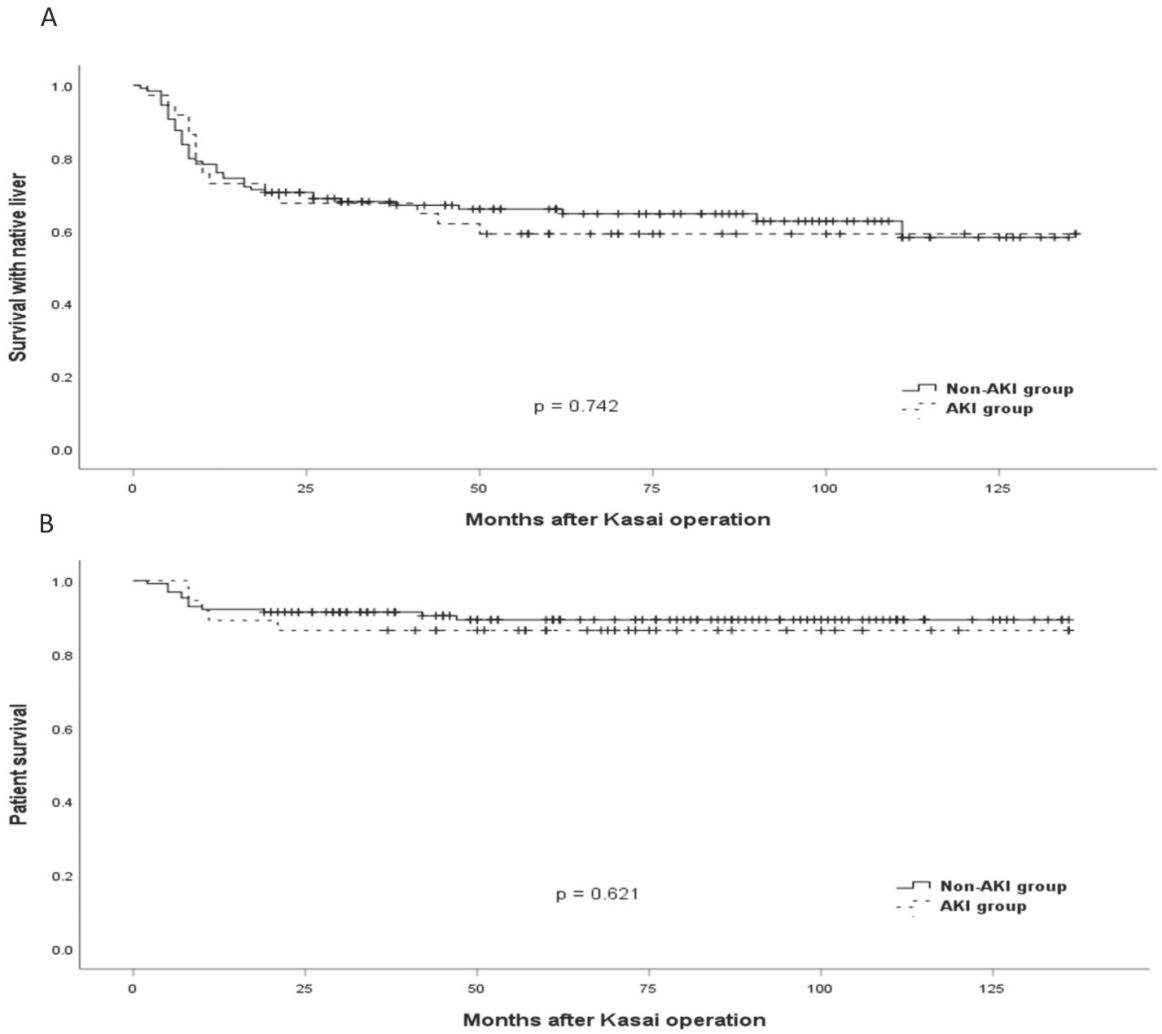
Time-to-Event curves for survival. (A) Comparison of the survival with native liver between patients with acute kidney injury (AKI) and without AKI. (B) Comparison of patient survival between patients with AKI and without AKI.

The comparison of cumulative SNL and patient survival between the AKI and the non-AKI groups is shown in Figure 1. There were no differences in SNL and patient survival between the two groups (*p* = 0.742 and 0.621, respectively).

## Discussion

In this retrospective study, which included 166 Kasai operation patients in single center, the incidence of AKI was 22.3%. Additionally, this study showed that age>30 days, higher preoperative eGFR, and preoperative contrast use within 7 days were associated with development of AKI. However, perioperative pRBC transfusion was inversely related to the occurrence of AKI. With respect to effect on outcome, AKI was associated with longer ICU stay and increased incidence of additional surgery apart from liver transplantation within 1 year. However, it did not affect outcomes such as SNL or patient survival.

As in adults, AKI is a well-known poor prognostic factor in children.^2^,^17^,^18^ Even a small postoperative increase in serum creatinine may have an impact on poor outcome in both children and adults.^4^,^5^ Considering that the epidemiology of AKI in children is completely different from that in adults,^1^ understanding AKI in children is important because ascertaining risk factors for AKI and early risk stratification may lead to early intervention. However, many previous studies have mainly focused on children who had cardiac surgery or critically ill children,^1^,^17^,^19^ not children who had other surgical procedures. In conducting this study, our hypothesis was that patients with biliary atresia are at high risk of AKI because they are much younger than other patients,^10^ have consequently immature kidney function,^20^ have higher bilirubin level associated with renal damage or hepatorenal syndrome,^21^ and have lower jaundice clearance, which was related to the development of AKI.^21^ This is supported by the study from Zhang et al., which demonstrated that biliary atresia was associated with an increased risk of AKI in children undergoing liver transplantation.^21^

In this study, we found that patients with age >30 days were more likely to develop AKI (*p* = 0.041). Our patients had an overall median age of 63 days: group median ages were 73 days in the AKI group and 58 days in the non-AKI group. This result is consistent with that of a study of 695 patients who underwent Kasai operation in France, which reported a median age of 60 days.^10^ However, previous studies of children who had cardiac surgery reported that younger age is a risk factor for the development of AKI.^18^,^22^ In those studies, immature glomerular development and nephrogenesis at the time of surgery were thought to contribute to the occurrence of AKI. This is inconsistent with our findings, and the possible explanation for this is that in patients who underwent Kasai operation, increasing age had deleterious effects on the course of biliary atresia. Serinet et al.^10^ and Schreiber et al.^23^ found that increased age at Kasai operation was associated with worse SNL outcome, especially in patients operated on after the age of 30 days. In that regard, there have been repeated but unsuccessful attempts to diagnose biliary atresia and perform surgery within the first month of life, while checking the color of stools.^10^ Therefore, the older the patient at the time of operation, the greater the progression of disease and development of complications, such as liver fibrosis or cholangitis, which may lead to the development of AKI. This is supported by the fact that direct bilirubin, which may reflect disease progression, was significantly higher in the AKI group than in the non-AKI group (6.6 vs 5.8 mg/dL, *p* = 0.035). Additionally, although there is no statistical significance, PELD score and APRi were higher in the AKI group. Therefore, this study confirmed the importance of early diagnosis and timing of operation of biliary atresia in reducing the risk of post-operative AKI.

Higher preoperative eGFR was associated with higher incidence of AKI in this study (*p* <0.001). In addition, preoperative serum creatinine was significantly lower in the AKI group (*p* <0.001). This is consistent with the results of previous studies, which showed that higher estimated creatinine clearance and lower serum creatinine are associated with higher incidence of AKI in children who had cardiac surgery.^2^,^18^ Lower serum creatinine (and hence, higher eGFR) is an important risk factor for AKI in children because it may be a reflection of lower muscle mass and malnutrition, or artifactual decrease due to fluid overload.^2^ In addition, higher eGFR contributes to increased exposure of the kidneys to nephrotoxins during the perioperative period.^2^ These reasons may explain why higher preoperative eGFR was associated with higher risk of AKI in this study.

Preoperative contrast use within 7 days before surgery was also associated with increased risk of AKI. In the AKI group, 89% of patients used contrast preoperatively within 7 days, whereas 79.8% used it in the non-AKI group. Contrast media are one of the leading causes of AKI because they have vasoconstrictive effects on renal blood vessels and direct renal tubular toxicity due to the high osmolality of some contrast agents.^24^ In our patients, contrast use within 7 days before surgery increased the incidence of AKI by more than five times (OR = 5.153, *p* = 0.044). Although contrast use is inevitable in most biliary atresia patients, it is a potentially modifiable risk factor among other causes of AKI. Therefore, further studies are needed to find ways to reduce AKI by adjusting use of contrast before surgery, because there is no simple method yet to prevent contrast-induced nephropathy in children.

Transfusion of pRBC was considered as a risk factor for AKI.^25^ However, perioperative pRBC transfusion was associated with decreased incidence of AKI in this study (OR = 0.284, *p* = 0.014). Possible explanation for this is that perioperative anemia, like blood transfusion, is independently associated with the risk of AKI.^26^,^27^ Karkouti et al. suggested the mechanisms by which perioperative anemia causes AKI in adults: the kidney is vulnerable to reduced oxygen delivery and renal hypoxia, and increased oxygen consumption in anemic patients leads to increased susceptibility to renal insults.^27^ In this study, the median preoperative hemoglobin level was 10.7 and 10.5 mg/dl in the AKI and the non-AKI group, respectively, both of which were slightly lower than the normal range. The lowest hemoglobin level showed a tendency to be lower in the AKI group at 24 h postoperatively, although there was no statistical significance (8.5 vs 8.9 mg/dL, *p* = 0.135). Therefore, correcting perioperative anemia and optimizing oxygen delivery by pRBC transfusion might have reduced the burden of AKI in our patients.

AKI was associated with longer duration of ICU admission in this study (OR = 1.015, *p* = 0.016). More patients in the AKI group also received additional surgery apart from liver transplantation within 1 year (10.8 % vs. 2.3 %, p = 0.045). This is consistent with the result of a previous study that AKI was associated with longer duration of ICU stay and hospital stay after cardiac surgery in children.^1^,^5^ Outcomes such as SNL and patient survival rates were comparable between the groups, but there was a trend towards decreased survival rates in the AKI group (59.5% vs. 64.3%, and 86.5% vs 89.9%, in the AKI and non-AKI groups, respectively). This is consistent with the result of a previous study that AKI did not affect graft survival and patient survival, but had a tendency with decreased survival in pediatric liver transplantation.^21^ Nevertheless, there were many previous studies that support a strong association between AKI and poor outcomes.^3^,^18^,^19^,^28^ Our findings of lack of association between AKI and prognosis might be the results of the lack of statistical power. Therefore, further studies are needed to investigate the association between AKI and long-term outcomes in children with biliary atresia.

We acknowledge that this current study has several limitations. First, AKIN urine criteria were not applied because data of hourly urine output were unavailable. However, a previous study showed that serum creatinine criteria have stronger association with the outcome than the urine output criteria, and that the addition of urine output criteria to serum creatinine criteria did not significantly affect the classification of AKI.^19^ There is also a study that was performed only with serum creatinine criteria, which investigated the incidence, risk factors, and outcomes of AKI after pediatric cardiac surgery.^1^ Second, there is an inherent limitation of a retrospective, single-center study, especially since all the surgeries were performed by one surgeon. However, this may also be regarded as an advantage as it gives assurance of consistency of surgical approach; consequently, potential surgical factors that may affect the occurrence of AKI can be excluded. Finally, the sample size was small because biliary atresia is a rare disease. Therefore, the results of this study should be interpreted with caution.

In conclusion, the development of AKI in biliary atresia patients after Kasai operation is associated with age >30 days, higher preoperative eGFR, preoperative contrast use within 7 days. Perioperative transfusion reduced the risk of AKI. AKI was related to longer ICU stay and increased incidence of additional surgery apart from liver transplantation within 1 year. Long-term outcomes, such as SNL and patient survival, appears to be worse in patients who developed AKI. These findings indicate a rational basis for early biliary atresia and AKI screening, and interventions to improve the results of Kasai operation, and deserve further study.

## Figures and Tables

**Table 1:** Patient demographics, perioperative, and postoperative outcomes according to the development of AKI.

	AKI (n=37)	Non-AKI (n = 129)	*P*-value
Preoperative			
age at surgery (days)	73 [59-88]	58 [41-78]	0.009
age >30 days (n)	36 (97.3)	106 (82.2)	0.021
weight (kg)	5 [4-6]	5 [4-6]	0.126
female (n)	25 (67.6)	75 (58.1)	0.302
prematurity (n)	9 (24.3)	22 (17.1)	0.317
kidney function assessment			
serum creatinine (mg/dl)	0.2 [0.2-0.2]	0.3 [0.2-0.4]	<0.001
eGFR (ml/min/1.73 m^2^)	126 [111-142]	86 [56-123]	<0.001
preoperative contrast use within 7 d (n)	33 (89.2)	103 (79.8)	0.193
hemoglobin (g/dl)	10.7 [9.9-11.7]	10.5 [9.7-11.7]	0.671
direct bilirubin (mg/dl)	6.6 [5.1-8.3]	5.8 [4.2-7.1]	0.035
PELD score	4.8 [3.3-5.9]	4.0 [2.6-6.2]	0.318
APRi	1.1 [0.6-2.4]	1.0 [0.6-1.7]	0.371
LSM	11 [8-17]	9 [6-16]	0.407
Intraoperative and postoperative for 48 h			
anesthetic time (min)	355 [330-390]	365 [330-395]	0.671
sevoflurane (n)	29 (78.4)	98 (76.0)	0.761
rocuronium/ atracurium (n)	31/6	105/24	0.739
blood loss (ml)	20 [8-50]	20 [10-38]	0.499
lowest systolic blood pressure (mmHg)			
intraoperative	50 [43-54]	49 [42-54]	0.748
postoperative 24 h	81 [76-91]	82 [73-90]	0.603
postoperative 48 h	91 [86-103]	89 [79-98]	0.352
lowest hemoglobin (mg/dl)			
intraoperative	9.5 [9.0 - 10.4]	9.3 [8.7 - 10.0]	0.116
postoperative 24 h	8.5 [7.8 - 9.8]	8.9 [8.1 - 10.0]	0.135
postoperative 48 h	9.9 [ 8.9 - 10.8]	9.9 [9.2 - 11.1]	0.389
pRBCs transfused (ml) /n	50 [0-65] /25	50 [13-69] /103	0.485/0.117
diuretics use (n)	7 (18.9)	18 (14.0)	0.457
vasoconstrictor use (n)	3 (2.3)	0	>0.999
number of antibiotics	3 [2.5-3]	3 [3-3]	0.189
length of ICU stay (h)	23 [20-46]	24 [21-42]	0.883
ventilation time (h)	0 [0-13]	0 [0-16]	0.345
Postoperative outcome			
length of hospital stay (days)	37 [34-44]	36 [34-41]	0.387
readmission within 1 year (n)	33 (89.2)	98 (76)	0.082
any additional surgery within 1 year (n)	4 (10.8)	3 (2.3)	0.045
liver transplantation (n)	11 (33.3)	40 (32.5)	0.930
death within 1 year (n)	4 (10.8)	10 (7.8)	0.516

Values are median (interquartile range), and number of patients (percentages). AKI, acute kidney injury; eGFR, estimated glomerular filtration rate; PELD, pediatric end-stage liver disease; APRi, aspartate aminotransferase-to-platelet ratio index; LSM, liver stiffness measure; pRBCs, packed red blood cells; ICU, intensive care unit.

**Table 2:** Multivariate logistic regression model of AKI after Kasai operation.

	Univariate			Multivariate			
Variable	p value	OR	95% CI	p value	OR	95% CI	
Age>30 days	0.060	18.486	0.885 - 386.3	0.041	22.711	1.142 - 451.8	
Preoperative eGFR (ml/min/1.73 m^2^)	0.001	1.019	1.008 - 1.031	0.001	1.020	1.008 - 1.032	
Preoperative contrast use within 7 days	0.051	4.920	0.991 - 24.4	0.044	5.153	1.046 - 25.4	
Direct bilirubin (mg/dL)	0.030	1.181	1.016 - 1.373				
Lowest systolic blood pressure (mmHg)	0.924	1.002	0.961 - 1.044				
Perioperative pRBC transfusion	0.013	0.282	0.103 - 0.768	0.014	0.284	0.105 - 0.773	
Length of ICU stay (h)	0.014	1.015	1.003 - 1.027	0.016	1.015	1.003 - 1.027	
Survival with native liver	0.588	0.813	0.384 - 1.719				
							

OR, odds ratio; CI, confidence interval; eGFR, estimated glomerular filtration rate; pRBC, packed red blood cells; ICU, intensive care unit.

## Abbreviations

AKI: acute kidney injury
AKIN: Acute Kidney Injury Network
APRi: AST-to-platelet ratio index
AST: aspartate aminotransferase
eGFR: estimated glomerular filtration rate
ICU: intensive care unit
OR: odds ratio
PELD: pediatric end-stage liver disease
pRBC: packed red blood cells
SNL: survival with native liver
